# NK Cell-Fc Receptors Advance Tumor Immunotherapy

**DOI:** 10.3390/jcm8101667

**Published:** 2019-10-12

**Authors:** Emilio Sanseviero

**Affiliations:** Immunology, Microenvironment and Metastasis Program, The Wistar Institute, Philadelphia, PA 19104, USA; esanseviero@wistar.org

**Keywords:** immunotherapy, NK cells, Fc receptors, combination therapies

## Abstract

Immunotherapy has revolutionized the treatment of cancer patients. Among immunotherapeutic approaches, antibodies targeting immune checkpoint inhibitors Programmed cell death protein 1 (PD-1) and cytotoxic T-lymphocyte-associated protein 4 (CTLA-4) are approved for treatment of metastatic melanoma and are in clinical trials for a variety of other cancers. The contribution of Natural Killer (NK) cells to the efficacy of immune checkpoint inhibitors is becoming more evident. Enhancing both T and NK cell function in cancer could result in a robust and durable response. Along with the ability to directly kill tumor cells, NK cells can mediate antibody-dependent cellular cytotoxicity (ADCC) given the expression of Fragment Crystallizable (Fc) receptors. Promising novel antibodies modified with improved Fc-receptor-mediated functions or Fc-engagers to kill target cells have been tested in pre-clinical models with considerable results. Combination therapies with immune-therapeutic antibodies with enhancers of NK-cell Fc-receptor-mediated function can be exploited to increase the efficacy of these antibodies. Herein, I discuss possible strategies to improve the success of immunotherapy by boosting NK cell function.

## 1. Introduction

**Figure 1:**
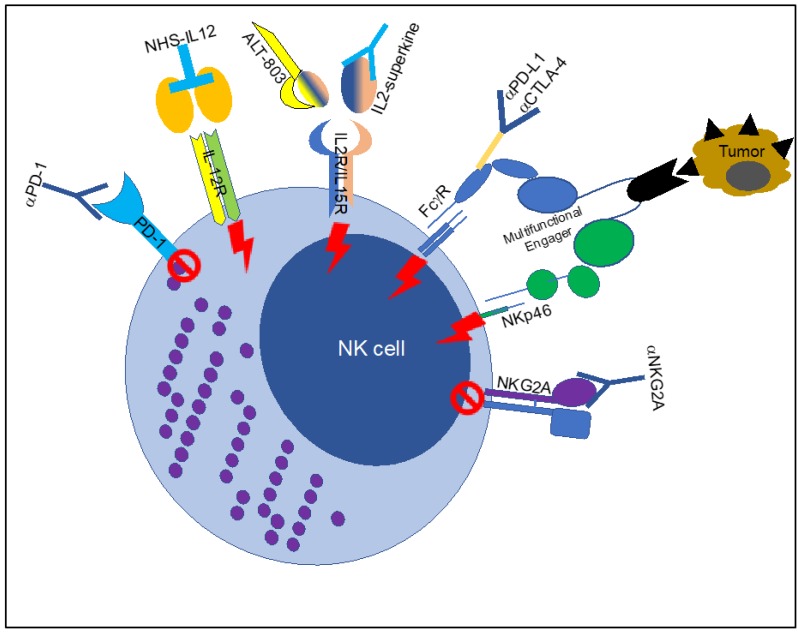
Multiple ways to trigger Fc-receptor function in NK cells. Schematic representation of the agents used to trigger Fc-receptor function in NK cells in the context of tumors. The figure includes the block of inhibitory receptors (mediated by anti-PD1 and anti-NKG2A or Natural Killer Group protein 2A) or the boosting of activating receptors (Interleukin (IL) 12 receptor, IL2/IL15 receptor, ADCC-enhanced antiPD-L1 or CTLA-4 and multifunctional receptor engagers). PD-1: Programmed cell death protein 1; ADCC: antibody-dependent cellular cytotoxicity; CTLA-4: cytotoxic T-lymphocyte-associated protein 4; PD-L1: Programmed death-ligand 1.

Natural killer (NK) cells are innate lymphoid cells and represent 5–20% of lymphocytes in human blood. These cells are characterized by a strong anti-tumor potential in terms of direct killing of cancer cells and immune regulation [1,2,3,4]. Intrinsic NK cell features make them particularly interesting for therapeutic intervention in cancer. Natural killer cells are engaged to kill target cells upon binding of ligands to activating receptors expressed on their cell surface [5,6,7,8,9,10,11,12]. In humans, one of the most efficient activating receptors expressed by NK cells is Cluster of Differentiation (CD) 16 or Fc γ Receptor (FcγR) IIIa [13,14,15,16,17,18,19,20,21]. The Fc receptors bind the Fc portion of the antibody and transduce activating or inhibitory signals into the cells [20]. The FcγRIIIa is the main Fc receptor expressed by human NK cells and induces activation signals and killing of target cells opsonized by the antibodies [21]. In some individuals, a fraction of NK cells can express FcγRIIc (CD32c), an inhibitory Fc receptor [22]. The study of Fc receptors is difficult because there is a divergence in human and mouse Fc receptor expression and function. Mouse FcγRIV seems to be the orthologue of FcγRIIIa, and mouse FcγRIII is the most closely related Fc receptor to human FcγRIIIa [21]. Murine NK cells, in homeostatic conditions, not only express FcγRIII but can also express FcγRIV in other conditions [21,23,24,25]. Immune checkpoint inhibitors (ICIs) are among the most efficient immunotherapeutic approaches currently used to treat cancer, and are antibodies that bind inhibitory molecules on the surface of tumor-infiltrating lymphocytes allowing anti-tumor immune responses to be reactivated [26,27,28,29,30,31,32,33]. In addition to this blocking ability, ICIs carry an Fc portion that elicits a separate biological effect resulting in the activation of Fc receptors [23,34,35,36,37,38,39,40,41,42,43,44]. In the tumor microenvironment, myeloid cells, monocytes, macrophages, neutrophils, and NK cells comprise the two main subsets of Fc-receptor expressing cells [23,34,35,37,40]. Often myeloid cells are deleterious and tumor-promoting, for this reason their stimulation should be carefully evaluated [45,46,47]. On the other hand, NK cell stimulation may potentially result in both enhanced Fc-mediated functions and increased direct tumor killing [48,49,50]. For this reason, boosting NK cells could represent a better option for combination therapy regimens (Figure 1).

## 2. Immune Checkpoint Inhibitors and NK Cell Fc Receptors

Antibodies targeting CTLA-4 and/or PD-1/PD-L1 are one of the most promising therapeutic approaches to treating cancer patients. PD-1 and CTLA-4 alone or in combination have been very successful and are approved for the treatment of metastatic melanoma and advanced PD-L1-positive non-small cell lung cancer (NSCLC) [51,52,53,54,55]. PD-1 is expressed by activated T cells and marks the so-called exhausted population of CD8 T cells and CD4. The signature of exhausted T cells represents a post-activation state of highly activated T-cells that undergo a state of anergy, becoming functionally inactive and, thus, unable to kill cancer cells or virus-infected cells [56]. PD-1 is expressed by NK cells, which is upregulated upon viral infection and tumor growth in both mice and human patients [57,58,59,60,61,62,63]. Antibodies targeting PD-1 release the break imposed on cytotoxic T cells and NK cells by the tumor microenvironment, thus allowing anti-tumor immune responses to be re-activated [63,64,65,66]. Anti-PD-1 antibodies in pre-clinical models showed poor Fc-mediated effects [67]. Human anti-PD-1 antibodies used in clinical practices include the human anti-PD-1 nivolumab and the humanized anti-PD-1 pembrolizumab. Both nivolumab and pembrolizumab are IgG4 and both are designed as blocking antibodies and, therefore, have poor Fc-mediated functions in order to avoid depletion of PD-1-expressing cells [68,69]. In fact, an ADCC-inducing version of anti-PD-1 has been shown to obstruct tumor clearance by depleting PD-1 expressing CD8 T cells and promoting tumor growth [67].

On the other hand, PD-L1 is expressed by tumor cells and immunosuppressive myeloid cells within the tumor microenvironment. Targeting these cells with ADDC-inducing antibodies can increase the efficiency of anti-PD-L1 antibodies [67]. Current anti-PD-L1 antibodies that are undergoing clinical trials or have been approved include atezolizumab, a fully humanized antibody; avelumab, a human antibody; and durvalumab, another human antibody. All three carry IgG1 Fc portions making them possible mediators of ADCC [70,71,72,73,74,75]. Murine pre-clinical models showed that anti-PD-L1 antibodies that carried the enhanced ADCC-inducing Fc portion IgG2a showed greater anti-tumor effects as compared to poorly ADCC-inducing version of PD-L1 [67]. The authors also showed that IgG2a-anti-PD-L1 was able to reduce immunosuppressive myeloid cells called myeloid-derived suppressor cells (MDSCs) and tumor-associated macrophages (TAMs) in the tumor microenvironment. Reduction of MDSC and TAM was attributed to reduced tumor growth. In fact, MDSC and TAM in tumors showed the highest levels of PD-L1 expression as compared to myeloid cells in the spleen or tumor cells themselves, making them ideal targets of ADCC-induced death [41]. In this case, the subset of cells responsible for the ADCC induction remains to be clarified. Interestingly avelumab showed an ability to trigger the activation of NK cells through Fc receptors. Specifically, avelumab-coated tumor cells in vitro were able to activate NK cells resulting in the induction of cytokine production and the killing of opsonized tumor cells through ADCC [72,76,77]. 

Additional studies are necessary to further elucidate the contribution of NK cells in vivo to the efficacy of anti-PD-L1 antibodies in both human and murine pre-clinical models.

Anti-CTLA-4 antibodies are used alone or in combination with PD-1/PD-L1 targeting antibodies. CTLA-4 is an inhibitory receptor expressed by activated T cells in certain conditions as a post-activation marker. As for anti-PD-1 antibodies, anti-CTLA4 antibodies were designed to block inhibitory signals received from T cells by the tumor microenvironment, allowing anti-tumor immune responses to be reactivated [70,78,79]. 

Besides activated CD8 T cells and conventional CD4 (cCD4) T cells, CTLA-4 is constitutively expressed by regulatory T cells (Treg) and represents one of the signature molecules expressed during Treg differentiation [80,81]. Tregs are a subset of immunosuppressive CD4 T cells. In cancer, Tregs promote tumor growth by inhibiting anti-tumor immune response. Tregs ablation in murine models results in reduced tumor growth and metastatic spread [82]. Anti-CTLA4 antibodies exhibit a second mechanism of action required for their efficacy. They are able to induce ADCC of tumor infiltrating Tregs. Tregs infiltrating both murine and human tumors express the highest levels of surface CTLA4 making them the perfect target of ADCC-mediated killing. Treg depletion by anti-CTLA4 antibodies in mice is mediated by FcγRIV and has been attributed to monocytes and myeloid cells [23,34,35,37,38,40,42]. We recently demonstrated that in mice, tumor-infiltrating NK cells do express FcγRIV [23]. In vivo treatment of tumor-bearing mice with anti-CLTA4 induces NK cell degranulation within the tumor. Specifically, anti-CTLA4 activates the NK cells that express FcγRIV concomitantly with Treg depletion. This suggests that NK cells contribute to anti-CTLA4 efficacy by inducing Treg depletion. Moreover, we also found that patients with cutaneous melanoma that benefit from ipilimumab, express higher CD56 RNA levels within the tumor compared to patients who did not benefit from ipilimumab treatment. CD56 is a marker that identifies NK cells; the positive correlation between CD56 RNA in the tumor and the benefit from anti-CTLA4 may suggest that increased NK cell infiltration can enhance anti-CTLA4 efficacy in humans [23]. Interestingly, in a murine preclinical model of melanoma where anti-CTLA4 treatment alone was not efficient, a combination of IL2 superagonist and anti-CTLA4 led to reduced tumor growth and this effect was abolished when NK cells were depleted [83].

Thus, NK cells play a pivotal role in the efficacy of ADCC-inducing therapeutic antibodies in cancer. This led to further studies aimed to test more antibodies able to induce ADCC of Tregs within the tumor microenvironment including anti-CD25 and anti-GITR antibodies. The Treg depletion reduced the immunosuppression within the tumor microenvironment resulting in a reduction of tumor growth [84,85,86].

## 3. Enhancing Therapeutic Antibodies for Cancer Treatment by Fc Engineering

The blocking function of antibodies is mediated by their antigen-binding fragment (Fab) portion. The Fab portion binds to a specific antigen thereby inhibiting the interaction of the antigen with other proteins, ultimately hindering its function. Antibodies also induce other effects mediated by the Fc portion. Among these effects, they can bind to Fc receptors expressed predominantly by myeloid or NK cells [20,21]. With protein engineering, it is now possible to generate antibodies with different Fc portions or different glycosylation patterns compared to the original antibody [87]. This is an extremely important attribute which can improve the efficacy of certain antibodies. Changing the IgG class or altering the glycosylation pattern of the antibodies can increase their affinity for some Fc receptors and decrease their affinity to others. Since both activating and inhibitory Fc receptors exist, making an ADCC-inducing antibody with less affinity for inhibitory receptors and greater affinity for activating receptors should potentiate its activity.

The study of ADCC is complicated by the differences existing in murine and human Fc receptors. In mice, ADCC is primarily mediated by FcγRIV, while in humans ADCC is predominantly exerted through FcγRIIIa. Moreover, the antibody isotypes that better induce ADCC in mice and humans are different. Murine IgG2a is the best ADCC inducer antibody isotype, while in humans IgG1 is the best ADCC inducer [20,21,24,25].

As discussed above, anti-CTLA4 activity relies, at least partially, on Treg depletion. Selby and colleagues [36] showed that the simple engineering of anti-CTLA4 antibody to express the IgG2a isotype drastically increased the efficacy of anti-CTLA4-treatment in terms of tumor growth and tumor eradication. This effect was due to the significant reduction of Tregs infiltration into the tumors [36]. Furthermore, different versions of anti-CTLA4 antibody with enhanced activating-Fc binding capabilities showed increased anti-tumor responses [37,38,42]. A new version of an anti-CTLA4 antibody with an enhanced ability to induce ADCC (BMS-986218) is now in clinical trial as a monotherapy or in combination with nivolumab (clinical trial identifier: NCT03110107). This antibody is modified in its glycosylation pattern to improve binding to activating Fc receptors.

Antibody glycosylation patterns affect the binding ability of antibodies to Fc receptors. Of particular interest for therapeutic antibodies is the N-terminal glycosylation of the human IgG, which are attached to Asn297 in the Cγ2 domain. This glycan contains a conserved pentasaccharide core of N-acetylglucosamine (GlcNAc) and mannose, plus other sugars ramifying the structure. A fucose is attached to the pentasaccharide core in most of the IgG. Removal of the fucose group to generate the so-called non-fucosylated antibody renders them more affine to the activating Fc receptor FcγRIIIa, thus promoting an enhanced ADCC [88,89,90].

One of the first non-fucosylated antibodies to enter into clinical use for cancer treatment is obinutuzumab, which was approved in the US in 2013. Obinutuzumab is a non-fucosylated anti-CD20 antibody that exhibited increased efficacy in patients with chronic lymphocytic leukemia compared to its ancestor rituximab, which targeted the same antigen but had lower ADCC-induction ability [91,92].

A second non-fucosylated antibody called mogamulizumab was approved in Japan in 2012 for CCR4+ cutaneous T cell lymphoma and was approved in 2018 in the US for certain forms of cutaneous T cell lymphomas. This antibody targets and induces ADCC of CCR4-expressing T cell lymphomas [93,94,95].

Both mogamulizumab and obinutuzumab opened the door to a new branch of science called glyco-engineering and show significant potential to be the future for antibody-based therapeutics. As such, a glyco-engineered anti-PD-L1 antibody has demonstrated improved ADCC capability in vitro compared to the original PD-L1 antibody and could possibly give even better results than the original PD-L1 antibody in vivo [96].

## 4. Activation of NK Cell Fc Receptors with Tumor-Targeting BI-Specific Antibodies and Bi-Specific or Tri-Specific Killer Engagers (Bikes, Trikes)

Chimeric antigen receptor expressing (CAR) T cells and NK cells have been reported to be extremely successful in some hematologic cancers, but it has been limited by several factors such as the high production cost, the time-consuming procedure to generate the treatment, and the high toxicity for patients. Bi-specific antibodies, BiKes, and the next-generation TriKes, Tri-engagers, offer a solution to the aforementioned hurdles, including reduced cost, less toxicity, and require less time-consuming preparation. These molecules are designed to create an immunological synapse between NK and tumor cells by concomitant engagement of at least one NK cell activating receptor and a tumor antigen to promote the lysis of the tumor by NK cells. These molecules are not usually generated using the full-length antibody, but rather only a single chain variable fragment (scFv) that contains the tumor antigen recognition site, which is connected to a second or to two other scFvs that recognize one or two NK cell activating receptors or to a linker that carries an NK cell activating cytokine.

Multiple factors make NK cells the most suitable target for bi-specific or tri-engager antibodies, including their innate killing ability and the expression of CD16, which is a potent NK cell activating receptor and which is already successfully exploited by ADCC-inducing antibody [48,97,98].

AFM13 is a bi-specific antibody from Affimed (Germany) targeting CD16 and CD30. It is currently in phase II clinical trial (clinical trial identifier NCT02321592) for the treatment of Hodgkin’s lymphoma. The results of the phase I trial have been published and showed the ability of the antibody to induce NK cell activation, reduction of CD30 in the blood, and an overall disease control rate of 61.5% [99].

A fusion molecule has also been generated that contains a CD16-binding scFv and NKG2D recombinant protein [100]. The NKG2D binds different stress-induced ligands that are expressed by tumor cells and not by normal cells, including acute myeloid leukemia [101]. This strategy is particularly innovative and seems to give good results in vitro, and can be used as a scaffold to generate multi-functional engagers or used in different types of cancer that express NKG2D ligands [100].

61533-Trike is a tri-functional recombinant molecule that has been generated for the treatment of myelodysplastic syndrome (MDS). It engages with CD16 on NK cells, CD33 on MDS cells, and carries a third arm of the molecule containing an IL15 cytokine moiety that promotes NK cell activation and function. This TriKe also allows NK cells to overcome immune inhibition mediated by immunosuppressive myeloid cells, further promoting anti-tumor immunity [102].

Of particular interest and remarkably promising is a recently generated multifunctional natural killer cell engager that targets CD16, a second NK cell activating receptor called NKp46, and a tumor antigen. This tri-engager has been constructed in three different versions in order to recognize three different tumor antigens. Thus, the use of this tri-engager can be particularly broad and can be virtually modified in order to recognize antigens expressed by different tumors. Specifically, the authors tested a tri-engager containing an anti-CD20, anti-CD19 or an anti-EGFR. Both the anti-CD20 and the anti-EGFR containing tri-engagers in vitro showed increased anti-tumor abilities compared to rituximab or obinutuzumab (a clinically used anti-CD20 antibody) or cetuximab (a clinically used anti-EGFR antibody) [103]. 

Of note, a simplified, long-lasting, and less expensive method for delivering bi-specific antibodies has been described and could represent a clever way to deliver TriKes and tri-engagers as well. This method is based on a DNA-encoded BiKe inserted into a plasmid that is delivered in vivo through adaptive electroporation. This delivery method will reduce the production costs and is less aggressive because a single delivery is long-lasting and has effective anti-tumor properties [104].

## 5. Stimulating NK Cells to Improve the Efficacy of Therapeutic Antibodies in Cancer

Different strategies have been described to improve NK cell efficacy against tumors. Combination therapy with therapeutic antibodies and potent immune cell activators represent a promising way to achieve a better response against cancers. Natural killer cell stimulating cytokines could potentiate the effect of checkpoint inhibitors, and blocking antibodies targeting NK cell inhibitory receptors could synergize with standard checkpoint blockades and induce potent anti-tumor immune responses.

Among NK cell stimulating cytokines, the three best described cytokines that are able to potentiate the activity of checkpoint inhibitors are IL2, IL15, and IL12.

The first cytokine described to enhance NK cell activity was IL2 and, to date, is the only Food and Drug Administration (FDA) approved cytokine for the treatment of cancer patients. However, IL2 has a big disadvantage in cancer as it targets Tregs, which are the cells that constitutively express the highest affinity (αβγ) receptor for this cytokine, leading to their expansion and, thus, increased tumor suppression. Despite the adverse effects, high doses of IL2 have been used to treat melanoma and renal cell carcinoma with positive results. In this case, the high doses bypassed the preferential effect on Tregs and, instead, activated effector T cells and NK cells stimulating an anti-tumor immune response [105,106].

To avoid the preferential effect on Tregs, IL2 has been used in a different form: complexed with an antibody. The IL2-Ab complexes (IL2-superkine) were shown to be have a lower affinity for the receptor expressed by Tregs and act preferentially on effector T cells [107,108].

Of note, the IL2 cytokine and the IL2-modified version have been described as being able to synergize with anti-CTLA4 and anti-PD1/PDL-1 antibodies in terms of tumor control, but only a combination of the IL2-based cytokine with CTLA4 is able to re-activate NK cells in vivo [83]. A possible explanation is that the increased activation markers expressed by NK cells and the increased NK/Treg ratio that resulted from the combination therapy are dependent on NK-cell killing of Tregs opsonized by anti-CTLA4 via ADCC. 

Indeed, NK cells have been shown to contribute to the efficacy of anti-PD-1/PD-L1 treatment through reinvigoration of NK cell effector functions [63]. 

IL15 is a second γ-chain cytokine that uses the same receptor as IL2 (β and γ chains). The high-affinity IL15 receptor α chain is expressed by IL15-producing cells, including dendritic cells, and inside their cytoplasm it is complexed with the cytokine and then trans-presented to the target cells [105]. 

IL15 presents different advantages compared to IL2, including the preferential activation and expansion of memory CD8 and NK cells that promote tumor control with no-Treg expansion.

IL15 alone has been shown to synergize with both anti-PD1 and anti-CTLA4 or a combination of the two checkpoint inhibitors [109].

Given the biology of IL15, the recombinant cytokine alone has actually modest effects in vivo. For this reason, a more potent IL15 version has been generated by combining recombinant IL15 and recombinant IL15 receptor α [110,111,112]. The IL-15/IL15 receptor α complex showed synergy and improved anti-tumor effects when combined with anti-CTLA4 and anti-PD-L1 ICI [23,113].

Of note, the results of a clinical trial with ALT-803 (IL15/IL15Rα complex) combined with nivolumab gave promising results in terms of clinical response in patients with metastatic NSCLC [114].

IL12 or its potentiated version NHS-IL12 can be used to boost anti-tumor immunity in both murine pre-clinical models and in humans [115,116]. Even more interesting, IL12 seems to be able to increase NK-cell-mediated ADCC [76,117,118]. A combination of IL12 and ADCC-inducing anti-PD-L1 or anti-CTLA4 showed impressive results in pre-clinical murine cancer models, including in a glioma model [76,119].

Cytokines are not the only avenue available to boost NK cell activity in the context of cancer. A NK-cell-specific immune-checkpoint has been described: IL1R8 that dampens NK-cell-mediated anti-tumor immunity. So far, the possibility of targeting IL1R8 with an antibody has not been explored yet, but could represent an effective way to boost NK cell activity in cancer, especially in a combination setting [120].

A promising and more defined approach is the use of antibodies targeting NKG2A, a checkpoint inhibitor expressed by both effector T cells and NK cells. Anti-NKG2A showed impressive results both in murine pre-clinical models and in human clinical trials. It synergizes well with both anti-PD-L1 to unleash T cell immune response, but also potentiates NK cell ADCC induced by the anti-EGFR antibody cetuximab [121].

## 6. Concluding Remarks

Over the past decade, the field of tumor immunology has made impressive strides in finding novel therapeutic strategies for cancer patients. Specifically, monoclonal antibodies targeting the immune system have opened a new era for cancer treatment. The impressive body of research towards understanding the mechanisms of action of these antibodies have provided significant insight on how the Fc portion of an antibody can impact an immune response. It is now clear that the Fc region of therapeutic antibodies is one of the main drivers contributing to their clinical efficacy. Moreover, new fields of study are now emerging, including glyco-engineering which is able to modify the function of Fc portions. Among Fc-expressing cells, NK cells are an ideal candidate to target and boost the efficacy of therapeutic antibodies. Although NK cells are endowed with the innate ability to kill tumors, it is clear that a combination approach is necessary to more efficiently treat cancer patients. Furthermore, additional research is necessary to identify the best combinations with the least adverse effects. To date, the majority of the research has been aimed at enhancing T cell function in cancer and has, indeed, achieved outstanding results. However, an approach that simultaneously boosts both T and NK cells could represent better ways to treat cancer patients in the near future.
